# A SIRPα‐Fc fusion protein enhances the antitumor effect of oncolytic adenovirus against ovarian cancer

**DOI:** 10.1002/1878-0261.12628

**Published:** 2020-02-07

**Authors:** Yao Huang, Sai‐qun Lv, Pin‐yi Liu, Zhen‐long Ye, Huan Yang, Lin‐fang Li, Hai‐li Zhu, Ying Wang, Lian‐zhen Cui, Du‐qing Jiang, Fang‐yuan Hao, Hui‐min Xu, Hua‐jun Jin, Qi‐jun Qian

**Affiliations:** ^1^ Department of Biliary Tract Shanghai Eastern Hepatobiliary Surgery Hospital China; ^2^ Laboratory of Viral and Gene Therapy Shanghai Eastern Hepatobiliary Surgery Hospital China; ^3^ Shanghai Cell Therapy Engineering Research Center China; ^4^ Xinyuan Institute of Medicine and Biotechnology College of Life Science Zhejiang Sci‐Tech University Hangzhou China

**Keywords:** cancer immunotherapy, CD47, oncolytic virus, ovarian cancer, SIRPα

## Abstract

Oncolytic viruses armed with therapeutic transgenes of interest show great potential in cancer immunotherapy. Here, a novel oncolytic adenovirus carrying a signal regulatory protein‐α (SIRPα)‐IgG1 Fc fusion gene (termed SG635‐SF) was constructed, which could block the CD47 ‘don't eat me’ signal of cancer cells. A strong promoter sequence (CCAU) was chosen to control the expression of the SF fusion protein, and a 5/35 chimeric fiber was utilized to enhance the efficiency of infection. As a result, SG635‐SF was found to specifically proliferate in hTERT‐positive cancer cells and largely increased the abundance of the SF gene. The SF fusion protein was effectively detected, and CD47 was successfully blocked in SK‐OV3 and HO8910 ovarian cancer cells expressing high levels of CD47. Although the ability to induce cell cycle arrest and cell death was comparable to that of the control empty SG635 oncolytic adenovirus *in vitro*, the antitumor effect of SG635‐SF was significantly superior to that of SG635 *in vivo*. Furthermore, CD47 was largely blocked and macrophage infiltration distinctly increased in xenograft tissues of SK‐OV3 cells but not in those of CD47‐negative HepG2 cells, indicating that the enhanced antitumor effect of SG635‐SF was CD47‐dependent. Collectively, these findings highlight a potent antitumor effect of SG635‐SF in the treatment of CD47‐positive cancers.

## Graphical abstract

A SIRPα‐Fc fusion protein enhances the antitumor effect of oncolytic adenovirus against CD47‐positive ovarian cancer.

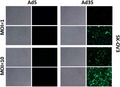

**Abbreviations**
Ad35: type 35 replication‐defective adenovirus
Ad‐blank: blank control replication‐defective adenovirus
Ad‐SF: replication‐defective adenovirus carrying a signal regulatory protein‐α (SIRPα)‐IgG1 Fc fusion gene
CAR: coxsackie adenovirus receptor
CCAU: a promoter
CMV: cytomegalovirus
MOI: multiplicity of infection
SF: SIRPα‐IgG1 Fc fusion gene
SG635: an oncolytic adenovirus
SG635‐SF: oncolytic adenovirus SG635 carrying a signal regulatory protein‐α (SIRPα)‐IgG1 Fc fusion gene
SIRPα: signal regulatory protein‐α

## 1 Introduction

CD47 is a cell‐surface transmembrane protein that is broadly expressed in normal tissues and functions as a ‘self’ signal by inhibiting phagocytosis through interaction with signal regulatory protein‐α (SIRPα) expressed on macrophages (Matozaki *et al.*, 2009; Oldenborg, 2004; Oldenborg *et al.*, 2000; Reinhold *et al.*, 1995; van den Berg and van der Schoot, 2008). This ‘self’ signal has been revealed to form part of the host immune‐escape strategy of malignant cells by high‐expressing CD47; that is, the ‘self’ signal is harnessed as a ‘don't eat me’ signal to allow the cancer cell to escape from the host immune system (Chao *et al.*, 2010; Edris *et al.*, 2012; Jaiswal *et al.*, 2009; Weiskopf *et al.*, 2013). Given its common high expression in malignant cancer cells, CD47 is associated with a poor clinical prognosis and metastasis and is thus considered a potential therapeutic target (Baccelli *et al.*, 2014; Majeti *et al.*, 2009; Wang *et al.*, 2013; Willingham *et al.*, 2012). Indeed, blocking CD47 with antibodies has shown a great therapeutic effect toward many malignances (Chao *et al.*, 2011; Edris *et al.*, 2012; Lee *et al.*, 2014; Sick *et al.*, 2012; Xiao *et al.*, 2015). However, blocking the binding between CD47 and SIRPα with antibodies has many drawbacks, including a limited tissue distribution and off‐target effects (Weiskopf *et al.*, 2013). Engineered SIRPα variants with higher affinity and specificity toward CD47 and a smaller size than wild‐type SIRPα, such as the engineered construct CV1, can bypass the limitation of tissue distribution and reduce the consequent side effects by antagonizing CD47; however, the higher affinity toward CD47 could exacerbate the off‐target effects (Ho *et al.*, 2015).

Oncolytic viruses are novel agents with recognized applications in cancer immunotherapy owing to their specific ability to selectively replicate in tumor cells and lyse targets via multiple mechanisms, including cytotoxic cytokine‐directed oncolysis, tumor vasculature targeting, and bystander effects (Bartlett *et al.*, 2013; Woller *et al.*, 2014). There has been rapid progress in the genetic manipulation of oncolytic viruses for clinical application (Pol *et al.*, 2014; Vacchelli *et al.*, 2013). The granulocyte–macrophage colony‐stimulating factor‐expressing oncolytic virus T‐VEC has been positively appraised, evaluated, and approved by the Food and Drug Administration owing to the great benefits conferred in patients with advanced melanoma in clinical trials. Nevertheless, there are still many factors hampering the therapeutic efficacy of oncolytic viruses, including blood component‐mediated virus neutralization, intratumoral stromal barriers causing inefficient intratumoral infiltration, and host immune system clearance (Smith *et al.*, 2011; Woller *et al.*, 2014). To overcome these issues, preclinical data suggest that the therapeutic combination of an oncolytic virus with other treatments would have a synergistic effect and optimize its therapeutic efficacy (Guo *et al.*, 2014; John *et al.*, 2012; Workenhe and Mossman, 2014).

Therefore, in this study, we genetically manipulated an oncolytic adenovirus designated SG635‐SF to express an engineered SIRPα and IgG1 Fc fusion protein and investigated its therapeutic effect against CD47‐high‐expressing ovarian cancer cells both *in vitro* and *in vivo*. These results can provide a novel therapeutic agent and strategy for CD47‐overexpressing cancer immunotherapy.

## 2 Materials and methods

### 2.1 Animals and cell culture

MRC‐5, HEK293, and HepG2 cells were purchased from the American Type Culture Collection (Manassas, VA, USA), and the ovarian carcinoma cell lines SK‐OV3 and HO8910 were purchased from the Institute of Cell Biology, Chinese Academy of Sciences (Shanghai, China). All cell lines were cultured according to the instructions from the providers in high‐glucose Dulbecco's modified Eagle's medium with 10% fetal bovine serum and incubated at 37 °C and 5% CO_2_.

Male Balb/c nude mice (16–20 g) were obtained from Slaccas Experimental Animal Co., Ltd. (Shanghai, China) and kept under standard conditions. Animal procedures were reviewed and approved by the Institutional Animal Care and Use Committee of Second Military Medical University.

### 2.2 Virus construction

We obtained fragments of the SIRPα‐IgG1 Fc fusion gene (hereafter SF) driven by the strongest promoter sequence (CCAU) found among several sequences tested (CCAU‐SF) by gene synthesis conducted at Shanghai (Generay Biotech Co., Ltd., Shanghai, China). CCAU‐SF and pCA16 (CN 201310460980.9) were digested by *Xba*I and *Sal*I, and then, the products were combined by ligase to generate the pCA16‐CCAU‐SF plasmid. CCAU‐SF‐SV40 PolyA, which was digested by *Bgl*II from the pCA16‐CCAU‐SF plasmid, and p74‐Tp (CN 201310460980.9), digested by the same enzyme, were reversely combined by ligase to generate the p74‐Tp‐CCAU‐SF plasmid. The purified p74‐Tp‐CCAU‐SF was cotransfected with PPE3‐F35, a plasmid of the type 35 adenoviral right arm, into HEK293 cells using the Lipofectamine^®^ 2000 kit (Invitrogen, CA, USA). Approximately 9–14 days after transfection, single virus plaques appeared in HEK293 cells, which were collected and confirmed by polymerase chain reaction analysis using the forward and reverse primers. The confirmed recombinant adenovirus was designated SG635‐SF. SG635‐SF was amplified in HEK293 cells, extracted using QIAamp DNA Blood Mini Kit (QIAGEN Inc., Dusseldorf, Germany), and purified by ultracentrifugation on cesium chloride (CsCl) gradients. Other viruses we used were treated with the same method. *EGFP* *gene* was cloned into the Ad5 and Ad35 to determine the infectivity.

Among several candidate promoter sequences, we selected that with the greatest amplification effect of the target gene using Lipo transfection reagent and the dual‐luciferase assay system (Promega, Madison, WI, USA). In brief, *Renilla* luciferase was controlled by the cytomegalovirus (CMV) promoter, firefly luciferase was controlled by various promoters, and the ratio of firefly to *Renilla* luciferase was calculated after cotransfection.

### 2.3 Viral replication *in vitro*

Cell lines were plated in six‐well dishes (10^6^ cells/well) and cultured until the cancer cells were in log phase and the normal cells appeared to exhibit contact inhibition and were infected with SG635 and SG635‐SF at a multiplicity of infection (MOI) of 5 PFU/cell. At 48 h after infection, the cells and supernatant were harvested and lysed by three freezing–thawing cycles. The titer data at 48 h were examined in HEK293 cells with the TCID50 method and normalized to virus production per cell based on the number of infected cells.

### 2.4 Western blot

Protein lysates from the cell lines were prepared in lysis buffer and centrifuged at 4 °C and 12 000 ***g***. Western blotting was performed according to a previously described procedure (Ye *et al.*, 2015) using rabbit anti‐human IgG1 (ABIN2862696), anti‐human CD47 (ab108415; Abcam, Cambridge, England), anti‐coxsackie adenovirus receptor (CAR; ab100811; Abcam, Cambridge, England), and anti‐CD46 antibody (ab108307; Abcam, Cambridge, England) as the primary antibodies. The secondary antibody was horseradish peroxidase‐labeled goat anti‐rabbit IgG (H+L) (Beyotime, Shanghai, China). The expression of each band was quantitatively analyzed using the image lab™ software (Bio‐Rad, CA, USA).

### 2.5 Flow cytometry

Cells were suspended and incubated with antibodies against CD47 (ab108415, Abcam, for SF protein blocking assay; and 556045, BD Bioscience, Franklin Lakes, NJ, USA, for other assays) and IgG1 on ice in the dark for 30 min, and then washed with normal saline twice and centrifuged for 5 min at 4 °C and 1500 ***g***. The cells were then analyzed by flow cytometry with the Guava system (Millipore, Burlington, MA, USA) and analyzed with Guava software.

### 2.6 Enzyme‐linked immunosorbent assay

Suspensions from SK‐OV3 and HO8910 cells were collected and diluted fivefold for SF protein secretion tests by ELISA using goat anti‐human IgG primary antibody, mouse anti‐goat secondary antibody, and cetuximab as a reference sample. Assays were carried out as previously described (Jin *et al.*, 2011).

### 2.7 Immunofluorescence assay

The cells were plated onto a confocal dish and fixed by paraformaldehyde for 20 min, followed by solubilization with Triton X‐100 for 10 min. The primary antibodies rabbit anti‐IgG1/CD47 were used at 1 : 100 dilutions, followed by incubation with fluorescein isothiocyanate‐labeled goat anti‐rabbit IgG (H+L) (Beyotime, Shanghai, China) secondary antibody. After immunolabeling, the cells were washed, stained with DAPI, and then scanned with a fluorescence microscope (Leica, Weztlar, Germany).

### 2.8 MTT assay

Cancer cells were cultured in 96‐well plates at 1 × 10^4^ cells per well. Twenty‐four hours later, the virus at the indicated MOIs was administered into cells. Seven days later, 50 μL of 3‐(4,5‐dimethylthiazol‐2‐yl)‐2,5‐diphenyltetrazolium bromide (MTT; 1 μg·mL^−1^) was added into cell media. Four hours later, MTT was discarded and 150 μL of DMSO was loaded in every well. The spectrophotometric absorbance of the samples was measured with Microplate Reader Model 550 (Bio‐Rad Laboratories, Hercules, CA, USA) at 570 nm with a reference wavelength of 655 nm. The percentage of cell survival was calculated using the following formula: cell survival = absorbance value of infected cells/absorbance value of uninfected control cells. Eight reduplicate wells were measured at each MOI, and every experiment was done at least three times.

### 2.9 Antibody‐dependent cytotoxicity assay

The EZ‐Cytox Cell Viability Assay Kit (ItsBio, Seoul, Korea) was used to measure the 4‐h cytotoxicity and antibody‐dependent cytotoxicity (ADCC) activities of cultured NK cells. Target tumor cells (4 × 10^4^/well) were seeded in a 96‐well flat‐bottom plate in triplicate and were cultured overnight under standard culture conditions. The next day, the target tumor cells were washed and exposed to media alone, 2.5, 10, and 40 μg·mL^−1^ purified SF protein at 37 °C for 30 min. After washing the cells twice, we cultured the cells with expanded murine NK cells at a 4 : 1 *E* : *T* ratio at 37 °C for 3 h. After adding 10 μL of WST‐1 (ItsBio, Seoul, Korea) to the well, the plates were incubated at 37 °C for 1 h and placed on ice for 5 min to stop the reaction. The absorbance at 450 nm was measured using the Infinite M200 PRO (Tecan, Männedorf, Switzerland). The percent of cytotoxicity was calculated using the following equation: 100% − 100 × [A450 of effector cell‐treated target cells − A450 of effector cells (background of effector cells)]/[A450 of target cells − A450 of target cells with no WST‐1 (background of target cells)].

### 2.10 *In vivo* assay

Balb/c nude mice (nu/nu) were purchased from Shanghai Experimental Animal Center, Chinese Academy of Sciences. All animal experiments were carried out in adherence to the National Institutes of Health Guidelines on the Use of Laboratory Animals and approved by the Navy Medical University (Second Military Medical University; Shanghai, China). Balb/c nude mice (nu/nu) were raised under the specific pathogen‐free (SPF) condition and housed under controlled temperature and humidity. For *in vivo* assessment of the antitumor effect of the oncolytic virus SG635‐SF, we subcutaneously transplanted SK‐OV3 cells into nude mice, allowed for xenograft formation, and then randomly divided the mice into five groups that were respectively treated by intratumoral injection of PBS, the replicative controls Ad‐blank and Ad‐SF, SG635, and SG635‐SF every day for a total of five times, and xenograft volumes were measured. In a separate experiment, the mice were injected with CD47‐positive HO8910 cells and CD47‐negative HepG2 cells to determine the dependency of the antitumor effect on CD47.

HO8910 and SK‐OV3 cells (1 × 10^8^ cells·mL^−1^) and control HepG2 cells (5 × 10^8^ cells·mL^−1^) were collected and suspended at a concentration of 1 × 10^8^ cells·mL^−1^ with PBS, incubated on ice, and subcutaneously injected into nude mice at 6–10 weeks of age. The mice were randomly divided into three groups (seven mice per group) when tumor tissues formed, and were then intratumorally injected with PBS, Ad‐blank, Ad‐SF, SG635, and SG635‐SF every day for five times in total, each time administering 2 × 10^8^ PFU virus in 100 μL PBS, respectively. Seven days after treatment, blood from the SK‐OV3‐transplanted mice was extracted for SF protein concentration assessment, and the xenograft volumes in cubic millimeters were measured as (*W* ^2^ × *L*)/2, where *W* is width and *L* is length.

### 2.11 Immunohistochemistry assay

All tumor tissues were collected from the above animals and fixed in 4% (w/v) paraformaldehyde, embedded in paraffin, and excised into 5‐mm samples. Following standard dewaxing procedures, the samples were stained with primary antibodies, including anti‐human CD47 (ab108415, Abcam, Cambridge, England), anti‐human Ki67 (Beyotime, Shanghai, China), and anti‐mouse CD68 (FA‐11, Abcam, Cambridge, England), anti‐mouse CD161c (MABF1495Z, Merck Millipore, MA, USA), anti‐mouse CD11b (ab133357, Abcam, Cambridge, England) and then were observed under a light microscope.

### 2.12 Statistical analysis

All data are presented as mean ± standard deviation. Independent Student's *t*‐test was used to analyze the variation between two selected groups, and Pearson's chi‐square test was used to analyze the correlation of two parameters. *P* < 0.05 was considered statistically significant, and *P* < 0.01 was considered highly statistically significant. All statistical analyses were performed with Statistical Package for the Social Sciences (spss) version 18.0 software (IBM, Chicago, IL, USA).

## 3 Results

### 3.1 Construction of the oncolytic adenovirus SG635‐SF

**Figure 1:**
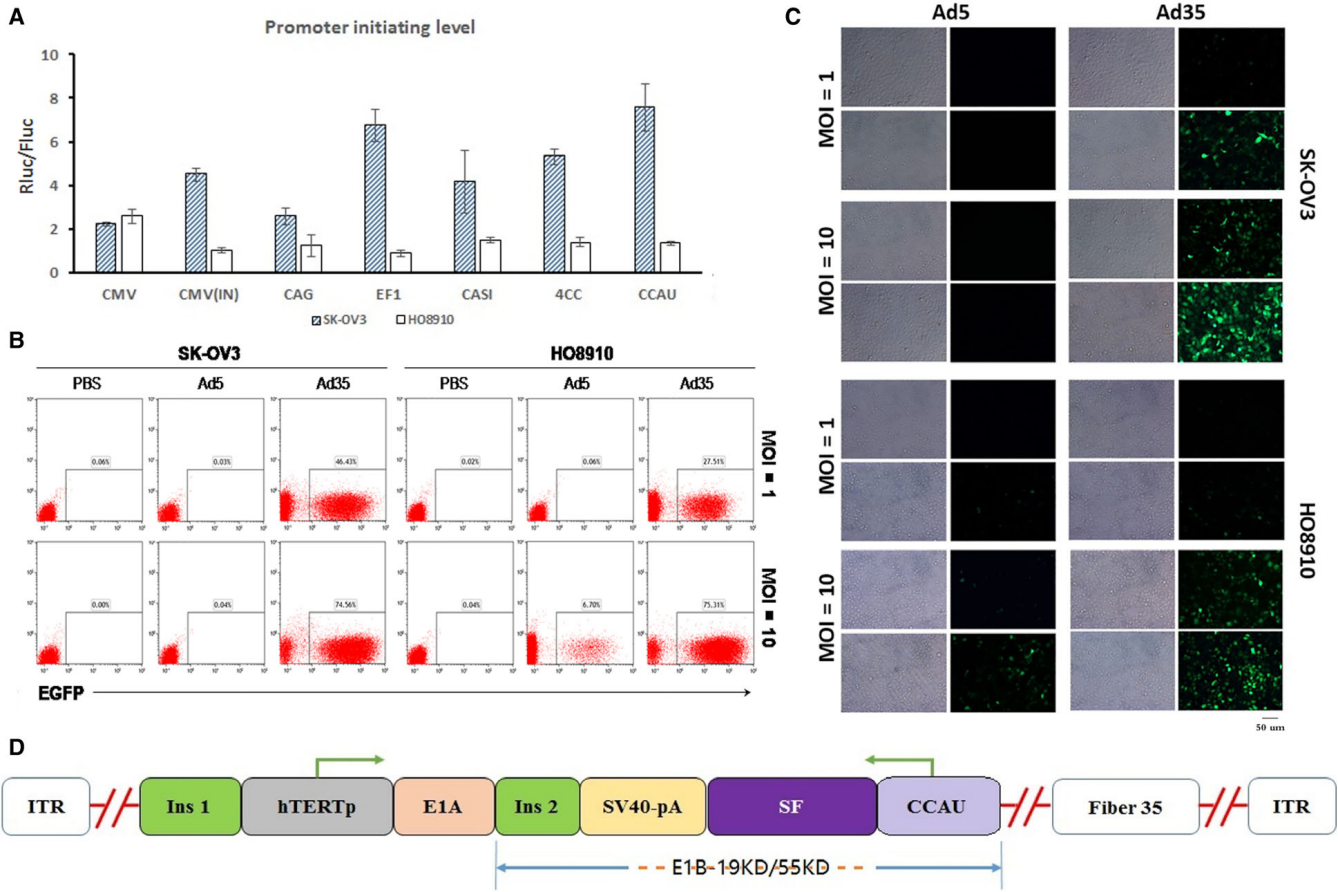
Construction of the oncolytic adenovirus SG635‐SF. (A) Expression initiation efficacy of promoters in SK‐OV3 (solid columns) and HO8910 (open columns) cells tested by a luciferase reporter system. Data are expressed as mean ± SD. (B) Flow cytometry was used to test the infectivity of Ad5 and Ad35 toward SK‐OV3 and HO8910 cells at a MOI of 1 and 10. (C) Fluorescence assay to confirm the infectivity of Ad5 and Ad35 toward SK‐OV3 and HO8910 cells at MOI = 1 and MOI = 10. Bar, 50 μm. (D) Linearized depiction of the plasmid for SG635‐SF.

Since the efficacy of the promoter in a plasmid is critical for the expression of target sequences and varies according to cell type, we used a luciferase reporter system to test the efficacy of a panel of promoters in CD47‐high‐expressing SK‐OV3 and HO8910 ovarian cancer cells. The results showed that CCAU, a novel designed chimeric promoter composed of a human CMV enhancer, mouse CMV enhancer, chicken β‐actin promoter, and ubiquitin intron in turn, was the optimal candidate given its ability to induce the greatest expression of the target gene in both SK‐OV3 and HO8910 cells at 48 h (Fig. 1A).

We then detected the expression of CD46 and CAR in SK‐OV3, HO8910, and HepG2 cells. The results showed that CD46 of these three cells lines were high‐expressed, indicating that they were more likely to be infected by Ad35‐type virus than Ad5‐type virus (Fig. S1). To improve the infection efficiency, a 5/35 fiber chimeric adenovirus (termed Ad35) was further adopted, which showed higher infection efficiency than type 5 adenovirus toward SK‐OV3 and HO8910 cells based on flow cytometry analysis (SK‐OV3: 46.43% *vs* 0.03%, 74.56% *vs* 0.04%; and HO8910: 27.51% *vs* 0.06%, 75.31% *vs* 6.70%) (Fig. 1B). Moreover, fluorescent microscopy observations further confirmed that Ad35 had higher infectivity than type 5 adenovirus (Fig. 1C).

The adenoviral shuttle plasmid vector containing the SF expression cassette driven by the CCAU promoter was constructed to obtain the recombinant oncolytic adenovirus SG635‐SF, within which the adenoviral E1A gene is under the control of the hTERT promoter. This limits replication of the virus within only hTERT‐positive tumor cells, as previously proven (Huang *et al.*, 2003; Su *et al.*, 2006). Subsequently, the adenoviral 19‐ and 55‐kDa coding genes of the E1B region were deleted and replaced by the SF expression cassette, thereby reducing adenoviral replication within the p53 wild‐type normal cells (Fig. 1D).

### 3.2 Oncolytic adenovirus SG635‐SF expresses SF and blocks CD47 expression

**Figure 2:**
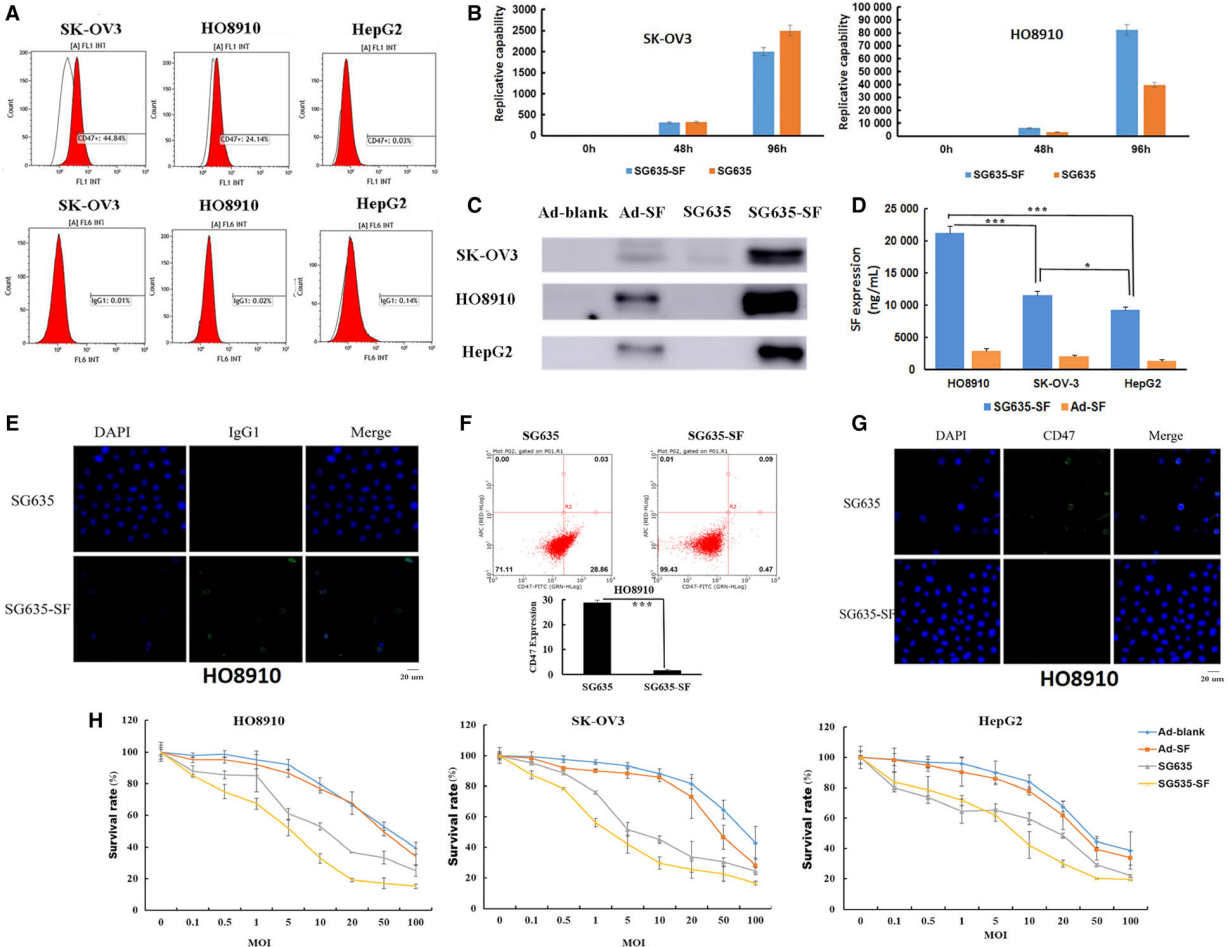
Confirmation of the successful construction of the oncolytic adenovirus SG635‐SF. (A) Flow cytometry to detect CD47 and IgG1 expression levels. (B) Replicative tests showing efficient replication of the oncolytic adenoviruses SG635 and SG635‐SF in both SK‐OV3 and HO8910 cells within 96 h. Data are expressed as mean ± SD. (C) Western blot for detecting the expression of SF protein in the cell lysate. (D) ELISA for detecting the expression of SF protein in the cell supernatant. Data are expressed as mean ± SD; Pearson's chi‐square test was used; ***P < 0.001; *P < 0.01. E: Fluorescence assay using FITC‐labeled anti‐IgG1 Fc antibody confirmed the expression of SF protein after oncolytic adenovirus SG635‐SF infection of HO8910 cells. Bar, 20 μm. (F, G) Flow cytometry and fluorescence assays revealed significant CD47 expression blockade of oncolytic adenovirus SG635‐SF compared to oncolytic adenovirus SG635. ***P < 0.001. Bar, 20 μm. (H) MTT assay showing the inhibitory effect of the oncolytic adenoviruses SG635 and SG635‐SF toward SK‐OV3 and HO8910 cells and HepG2 cells. Data are expressed as mean ± SD.

We confirmed that CD47 is positive in the ovarian cancer cell lines SK‐OV3 and HO8910, while it is almost negative in HepG2 cell line (Fig. 2A). The oncolytic adenovirus SG635‐SF could effectively replicate in SK‐OV3, HO8910, and HepG2 tumor cells postinfection while having lower replicative capability in MRC‐5 normal cell lines (Fig. 2B and Fig. S2). Western blot and ELISA showed that SF protein was detectable in both the cell lysates and supernatants of SK‐OV3, HO8910, and HepG2 cells (Fig. 2C,D). Moreover, binding of SF protein to the cellular membrane was further confirmed by an immunofluorescence assay using an antibody against human IgG1 Fc (Fig. 2E). Therefore, CD47 blockade on the cell surface was further investigated using immunofluorescence and flow cytometry assays. SF protein secreted by the infected tumor cells could bind with CD47 receptor on the cell surface and block the binding of anti‐CD47 antibody (ab108415) with it. The results showed that CD47 was successfully blocked in HO8910 cells infected with SG635‐SF when compared to those infected with the control oncolytic adenovirus SG635 (Fig. 2F,G). Collectively, these results indicated that SF protein could be effectively expressed to block CD47 when cancer cells are infected with SG635‐SF.

### 3.3 The oncolytic adenovirus SG635‐SF induces cell cytotoxicity

We next investigated the antitumor activity of the oncolytic adenovirus SG635‐SF. The MTT assay was applied to test the inhibitory effect of the oncolytic adenovirus SG635‐SF on cell viability of SK‐OV3, HO8910, and HepG2 cells on day 7. The results showed no distinct difference in the capacity of the oncolytic adenoviruses SG635‐SF and SG635 to inhibit the proliferation of SK‐OV3 and HO8910 cells; however, the inhibitory effect was more potent compared to that of the replication‐defective control Ad‐SF‐ and Ad‐blank‐infected groups at MOI = 20. Similar results were observed when the same adenoviruses were infected in CD47‐negative HepG2 cells (Fig. 2H).

### 3.4 The oncolytic adenovirus SG635‐SF inhibits xenograft growth *in vivo*

**Figure 3:**
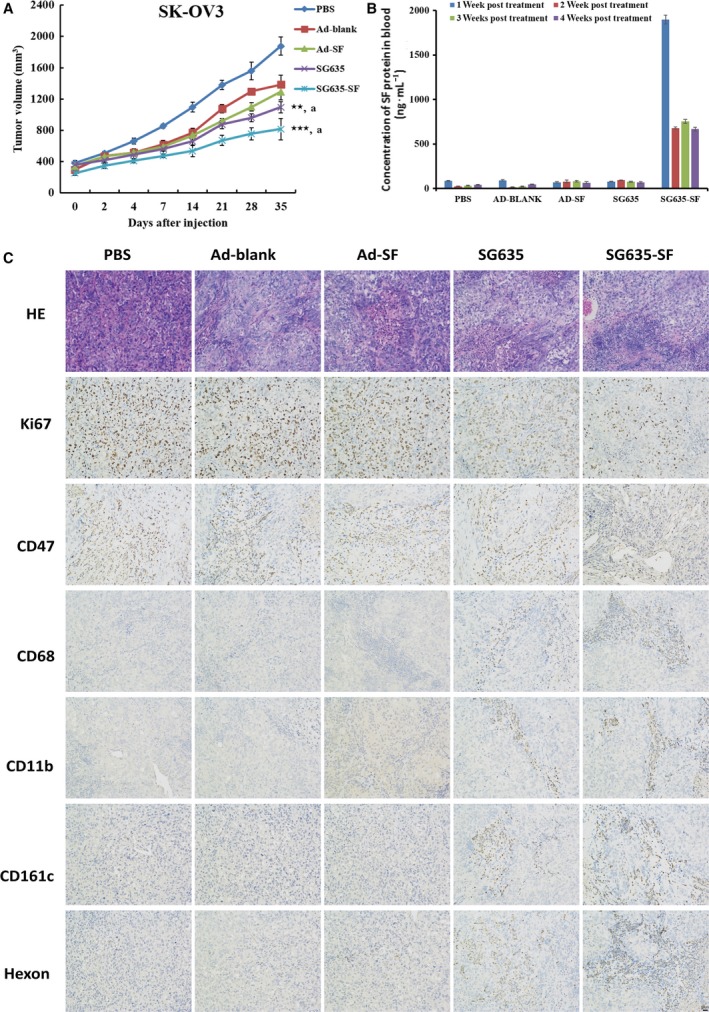
*In vivo* antitumor activity of the oncolytic adenovirus SG635‐SF. (A) Xenografts of mice treated with PBS, adenovirus Ad‐blank, Ad‐SF, SG635, and SG635‐SF. Values are presented as mean ± SD (*n* = 7). Pearson's chi‐square test was used; ***P < 0.001 *vs* Ad‐blank group; **P < 0.01 *vs* Ad‐blank group; ^a^ *P* < 0.001 *vs* PBS group. (B) ELISA for detection of the concentration of SF protein in the blood at 4 weeks post‐treatment. (C) Immunohistochemistry using anti‐Ki67, CD47, CD68, and Hexon, CD11b, CD161c antibodies revealed growth inhibition, CD47 expression blockade, macrophage‐mediated phagocytosis, and viral infiltration enhancement of the oncolytic adenovirus SG635‐SF compared to other treatments. Data are expressed as the mean ± SD.

Based on the promising results *in vitro*, we further investigated the antitumor activity of the oncolytic adenovirus SG635‐SF *in vivo*. Xenograft growth was inhibited in the two oncolytic adenovirus treatment groups (i.e., SG635‐SF and SG635) when compared with that of the replication‐defective control groups (i.e., Ad‐SF and Ad‐blank) and the placebo group (PBS). Interestingly, a significant difference was observed between the SG635‐SF and SG635 groups (*P* = 0.0096; Fig. 3A). However, no distinct difference was found between the Ad‐SF and Ad‐blank groups. We then detected the concentration of SF protein in the blood using ELISA, which showed that SF protein was detectable in the oncolytic adenovirus SG635‐SF group (1898.77 ng·mL^−1^), and the concentration of SF protein in the blood of SG635‐SF‐treated mice decreased after the second week post‐treatment (680.26 ng·mL^−1^) and persisted until the fourth week (668.52 ng·mL^−1^), but SF was barely detected in the other groups, including the Ad‐SF group (Fig. 3B). This result could partly explain the different situation between the two comparisons described above (i.e., SG635‐SF *vs* SG635 and Ad‐SF *vs* Ad‐blank) and suggested that higher concentration of SF protein could improve the antitumor capacity *in vivo*.

The immunohistochemistry assay was then used to survey the molecular difference among all treatment groups, indicating that the positively stained area of Ki67 and CD47 was greatly reduced in the SG635‐SF group compared to that of the other groups. However, the Hexon (indicating adenovirus particles) signal was clearly detected in both the SG635‐SF and SG635 groups, suggesting that there was no difference in viral replication between the two groups (Fig. 3C). Interestingly, infiltration of CD11b‐ and CD68‐positive macrophages and CD161c‐positive NK cells was improved in the xenograft tissue of the SG635‐SF treatment group, suggesting that the phagocytosis of macrophages was promoted owing to CD47 blockade (Fig. 3C). These results suggested that the oncolytic adenovirus SG635‐SF could more effectively inhibit the growth of SK‐OV3 xenografts through the synergistic effect of the oncolytic adenovirus and CD47 blockade.

### 3.5 The oncolytic adenovirus SG635‐SF inhibits ovarian cancer cell growth *in vivo* in a CD47‐dependent manner

**Figure 4:**
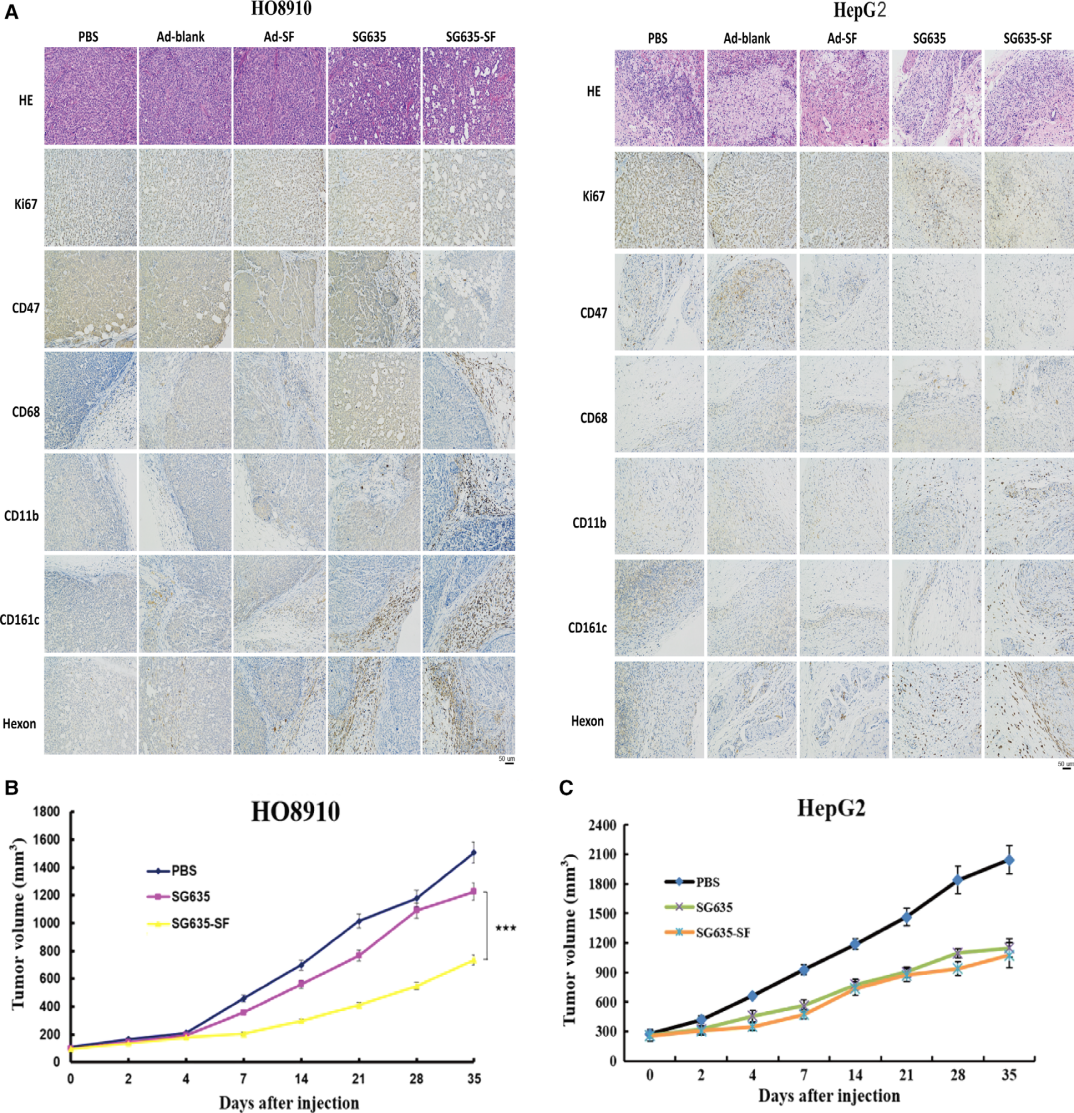
Antitumor activity of the oncolytic adenovirus SG635‐SF is CD47‐dependent. (A) Immunohistochemistry results revealed growth inhibition, CD47 expression blockade, macrophage‐mediated phagocytosis, and viral infiltration enhancement of the oncolytic adenovirus SG635‐SF compared to other treatments. (B) Oncolytic adenovirus SG635‐SF inhibited the growth of the CD47‐positive HO8910 cell‐derived xenografts more strongly than SG635. Values are mean ± SD (*n* = 7); Pearson's chi‐square test was used; ***P < 0.001 *vs* SG635 group. (C) The inhibition ability of oncolytic adenovirus SG635‐SF had no significant difference compared with the group of SG635 in CD47‐negative HepG2 cell‐derived xenografts. Values are mean ± SD.

HO8910 is a CD47‐positive cell line, and HepG2 is a CD47‐negative cell line. The results of treating these two kinds of tumor‐derived xenografts with the same method showed that SG635‐SF could block CD47 expression in HO8910 xenografts effectively and recruit relative murine immune cells, including macrophages and NK cells (Fig. 4A). But this phenomenon could not be observed in HepG2 xenografts. Treatment with the oncolytic adenovirus SG635‐SF significantly inhibited the growth of HO8910 cell‐derived xenografts when compared to the effect of SG635 or PBS (Fig. 4B). However, the inhibitory effect of SG635‐SF and SG635 was comparable in the HepG2‐derived xenografts (Fig. 4C). Ad‐blank and Ad‐SF could slightly inhibit tumor growth in both HO8910 and HepG2 animal models (results not shown). These results indicated that the antitumor effect of the oncolytic adenovirus SG635‐SF was CD47‐dependent. Considering that SF protein could mediate ADCC effect on CD47‐positive cancer cells *in vitro* (Fig. S3), the enhanced antitumor capacity of oncolytic adenovirus SG635‐SF on CD47‐positive xenografts might be due to both phagocytosis enhancement and ADCC effect via CD47 blockade by the SF protein.

## 4 Discussion

We demonstrated that the oncolytic adenovirus SG635‐SF armed with the SIRPα‐Fc fusion gene exhibited a great inhibitory effect on CD47‐positive cancer cells both *in vitro* and *in vivo*. However, no distinct difference was observed between SG635‐SF and SG635, or between the CD47‐positive and CD47‐negative cancer cells *in vitro*, suggesting that SF protein expression and consequent CD47 blockade had no effect on enhancing the antitumor capacity of an oncolytic adenovirus without other immune cells, such as macrophages. By contrast, a significant difference was detected between the same pairs *in vivo*, indicating that CD47 blockade successfully reinforced macrophage‐mediated phagocytosis or ADCC effect. Moreover, adenovirus proliferation in cancer cells could induce cell lysis and thus promote tumor antigen release while simultaneously triggering danger signals to recruit immune cells into the cancer tissue. Therefore, SG635‐SF had a synergistic effect through the oncolytic adenovirus and CD47 blockade, making it a potent candidate in treating CD47‐positive cancers.

To improve the expression of SF protein in cancer cells, we used the novel chimeric promoter CCAU to drive the transcription of the SF fusion gene and adopted the 5/35 chimeric fiber to enhance the infection efficacy. Merely, the autocrine secretion of SF fusion protein might quickly lead to CD47 blockade and subsequent phagocytosis. Thus, the progeny adenoviruses might not have sufficient time to be packaged and really reduce the expression of SF *in vivo*. Fortunately, the concentration of SF protein in the blood was found to peak on the 7th day following adenoviral injection, which lasted for 3 weeks, indicating that the adenoviral construction can effectively express SF protein *in vivo* as expected. However, a more optimal design should be further investigated so as to equilibrate the expression of SF protein and the proliferation of the adenovirus.

CD47 is broadly expressed in almost all cell types, including normal and malignant cells (Ye *et al.*, 2015). Engineered SIRPα variants with much higher affinity toward CD47 could overcome the issues of limited tissue distribution, but might unintentionally exacerbate the off‐target effects. Wild‐type SIRPα was proposed as the ideal CD47 blocking agent to reach a trade‐off between achieving a potent therapeutic effect and enhanced unwanted binding (Chen *et al.*, 2014). However, in this study, the engineered SIRPα variant was designed so as to combine with the oncolytic adenovirus owing to its high specificity for tumor cells and ability to lyse targets while sparing normal cells. Hence, to further survey the effect of this novel combination on other cancers toward its clinical application, further investigation of its precise targeting ability was needed.

Nevertheless, as the old proverb states, every coin has two sides. The ‘don't eat me’ signal achieved through high expression of CD47 is important for the survival of cancer cells and can also be harnessed to increase the longevity of target cells. In a recent study, CD47‐positive artificial antigen‐presenting cells were generated to highly express CD47, which were consequently endowed with a longer functional half‐life that facilitated the antitumor activity of their partners (Soto‐Pantoja *et al.*, 2012), demonstrating the other side of the ‘don't eat me’ coin. Thus, it is of great interest to investigate whether this aspect of the ‘don't eat me’ signal can also be helpful for other immunotherapies, including for the treatment of autoimmune diseases.

In addition to its function as a ‘don't eat me’ signal, CD47 controls cell differentiation and the stress response, along with regulating the state of pancreatic cancer stem cells (AACR Annual Meeting, 2014; Bruns *et al.*, 2015). Thus, further investigations are needed to reveal the underlying mechanisms of CD47 blockade for cancer treatment, such as the impacts of the oncolytic adenovirus SG635‐SF on the differentiation and stress response of target cells, its effect on cancer stem cells, and how to incorporate other important factors related to the targeting effects in the SG635‐SF design. Recently, CD47 blockade was shown to enhance antitumor immune responses (Soto‐Pantoja *et al.*, 2014). The increased expression of CD47 and the ‘don't eat me’ signal released by CD47 could be observed in many different human tumors, indicating that the CD47–SIRPα pathway is a potential target for treatment of human malignant tumors. By blocking the CD47–SIRPα pathway, macrophages can rapidly and effectively phagocytize tumor cells, thus promoting tumor inhibition (Chao *et al.*, 2011). Moreover, tumor cells can also be eliminated by blocking the CD47–SIRPα pathway through Fc‐dependent mechanisms, which include ADCC and complement‐dependent cytotoxicity effect (Johansson *et al.*, 2004). Our immunohistochemical results showed that the expression of SF protein in mice could effectively block the CD47–SIRPα pathway, recruit peripheral macrophages and NK cells, and kill tumor cells efficiently. Hence, oncolytic virus armed with SIRPα‐Fc fusion protein combined with other immunotherapies is promising in antitumor application.

## 5 Conclusion

In summary, our present study provided an oncolytic virus, SG635‐SF, carrying a SIRPα‐IgG1 Fc coding gene. Evidence showed the novel oncolytic virus was very promising in cancer immunotherapy, while arming with 5/35 chimeric fiber improved the efficiency of infection. Consequently, a strong enhanced antitumor effect of SG635‐SF was proved *in vivo* and the enhanced antitumor effect of SG635‐SF was CD47‐dependent, suggesting the potency of SG635‐SF in the treatment of CD47‐positive cancer.

## Conflict of interest

The authors declare no conflict of interest.

## Author contributions

QQ and HJ were responsible for coordination and manuscript editing. ZY and HY drafted the manuscript. SL, PL, LL, DJ, and HX conducted *in vitro* experiments. YH, HZ, and LC conducted *in vivo* experiments. FH and YW helped in data interpretation and statistical analysis. All authors had substantial contributions to the conception or design of the work, read the final manuscript, and agreed with the accuracy and integrity of all parts of the work.

## Supporting information


**Fig. S1.** The expression of CD46 and CAR in SK‐OV3, HO8910, and HepG2 cell lines.
**Fig. S2.** Replication efficiency of SG635 and SG635‐SF in HepG2 cells and MRC‐5 cells. Values are mean ± SD.
**Fig. S3.** ADCC of murine NK cells against SK‐OV3 cells and HepG2 cells (E:T ratio, 4:1) in the absence or presence of SF protein. Values are mean ± SD.
