# Supplementary material for: A SIRPα‐Fc fusion protein enhances the antitumor effect of oncolytic adenovirus against ovarian cancer
Source: Mol Oncol. 2020 Feb 7;14(3):657–68. doi: 10.1002/1878-0261.12628 (PMC7053234; doi:10.1002/1878-0261.12628)
Supplement: Supplementary file 1 — Fig. S1. The expression of CD46 and CAR in SK‐OV3, HO8910, and HepG2 cell lines. Fig. S2. Replication efficiency of SG635 and SG635‐SF in HepG2 cells and MRC‐5 cells. Values are mean ± SD. Fig. S3. ADCC of murine NK cells against SK‐OV3 cells and HepG2 cells (E:T ratio, 4:1) in the absence or presence of SF protein. Values are mean ± SD. [file MOL2-14-657-s001.docx]

Supporting Information for

**A SIRPα-Fc fusion protein enhances the antitumor effect of oncolytic adenovirus against ovarian cancer**

Yao Huang^a #^, Sai-qun Lv^b, c #^, Pin-yi Liu^d #^, Zhen-long Ye^b, c^, Huan Yang^c^, Lin-fang Li^b, c^, Hai-li Zhu^b^, Ying Wang^b^, Lian-zhen Cui^c^, Du-qing Jiang^c^, Fang-yuan Hao^c^, Hui-min Xu^c^, Hua-jun Jin^b, c *^, Qi-jun Qian ^b, c, d*^

*^a^ DepartmentⅠof biliary tract, Shanghai Eastern Hepatobiliary Surgery Hospital, Shanghai 200438, China*

*^b^ Laboratory of Viral and Gene Therapy, Shanghai Eastern Hepatobiliary Surgery Hospital, Shanghai 200438, China*

*^c^ Shanghai Cell Therapy Engineering Research Center, Shanghai 201805, China*

*^d^* *Xinyuan Institute of Medicine and Biotechnology College of Life Science, Zhejiang Sci-Tech University, Hangzhou 310018, China*

# These authors contributed to this study equally

** Correspondence authors:*

*Qi-jun Qian, Shanghai Engineering Research Center for Cell Therapy, Shanghai 201805, China; E-mail: qian@shcell.org*

*or Hua-jun Jin, Shanghai Engineering Research Center for Cell Therapy, Shanghai 201805, China; E-mail: hj-jin@hotmail.com*

**
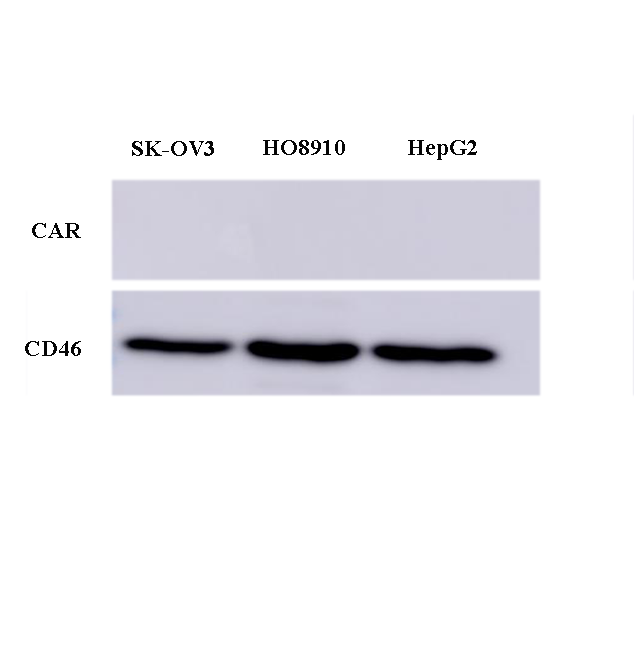
**

Fig. S1 The expression of CD46 and CAR in SK-OV3, HO8910, and HepG2 cell lines.

Fig. S2 Replication efficiency of SG635 and SG635-SF in HepG2 cells and MRC-5 cells. Values are mean ± SD.


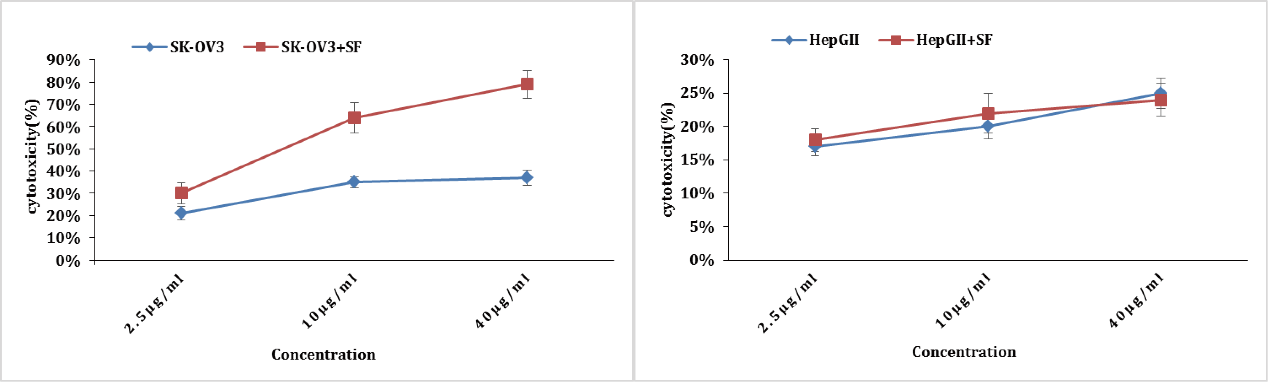


Fig. S3 ADCC of murine NK cells against SK-OV3 cells and HepG2 cells (E:T ratio, 4:1) in the absence or presence of SF protein. Values are mean ± SD.

SIRPα-Fc sequence is as follows:

ATGGAGTTTTGGCTGAGCTGGGTTTTCCTTGTTGCTATTTTAAAAGGTGTCCAGTGTGAGGAGGAGCTGCAGATCATTCAGCCTGACAAGTCCGTGTTGGTTGCAGCTGGAGAGACAGCCACTCTGCGCTGCACTATCACCTCTCTGTTCCCTGTGGGGCCCATCCAGTGGTTCAGAGGAGCTGGACCAGGCCGGGTGTTAATCTACAATCAAAGACAGGGCCCCTTCCCCCGGGTAACAACTGTTTCAGACACCACAAAGAGAAACAACATGGACTTTTCCATCCGCATCGGTAACATCACCCCAGCAGATGCCGGCACCTACTACTGTATCAAGTTCCGGAAAGGGAGCCCCGATGACGTGGAGTTTAAGTCTGGAGCAGGCACTGAGCTGTCTGTGCGCGCCAAACCCGGTGGAGGTGGAGGTGGAGGTGGAGGTCCTAAATCTTGTGACAAAACTCACACATGCCCACCGTGCCCAGCACCTGAACTCCTGGGGGGACCGGACGTCTTCCTCTTCCCCCCAAAACCCAAGGACACCCTCATGATCTCCCGGACCCCTGAGGTCACATGCGTGGTGGTGGACGTGAGCCACGAAGACCCTGAGGTCAAGTTCAACTGGTACGTGGACGGCGTGGAGGTGCATAATGCCAAGACAAAGCCGCGGGAGGAGCAGTACAACAGCACGTACCGTGTGGTCAGCGTCCTCACCGTCCTGCACCAGGACTGGCTGAATGGCAAGGAGTACAAGTGCAAGGTCTCCAACAAAGCCCTCCCAGCCCCCGAGGAGAAAACCATCTCCAAAGCCAAAGGGCAGCCCCGAGAACCACAGGTGTACACCCTGCCCCCATCCCGGGATGAGCTGACCAAGAACCAGGTCAGCCTGACCTGCCTGGTCAAAGGCTTCTATCCCAGCGACATCGCCGTGGAGTGGGAGAGCAATGGGCAGCCGGAGAACAACTACAAGACCACGCCTCCCGTGCTGGACTCCGACGGCTCCTTCTTCCTCTACAGCAAGCTCACCGTGGACAAGAGCAGGTGGCAGCAGGGGAACGTCTTCTCATGCTCCGTGATGCATGAGGCTCTGCACAACCACTACACGCAGAAGAGCCTCTCCCTGTCTCCGGGTAAA
